# The cost-effectiveness of B-type natriuretic peptide-guided care in compared to standard clinical assessment in outpatients with heart failure in Tehran, Iran

**DOI:** 10.1186/s12962-021-00334-z

**Published:** 2021-12-23

**Authors:** Aziz Rezapour, Andrew J. Palmer, Vahid Alipour, Marjan Hajahmadi, Abdosaleh Jafari

**Affiliations:** 1 Health Management and Economics Research Center, Iran University of Medical Sciences, Tehran, Iran; 2 Department of Health Economics, School of Health Management and Information Sciences, Iran University of Medical Sciences, Tehran, Iran; 3 Menzies Institute for Medical Research, University of Tasmania, Hobart, Tasmania Australia; 4 Centre for Health Policy, School of Population and Global Health, University of Melbourne, Melbourne, Victoria Australia; 5 Cardiologist, Fellowship in Heart Failure and Cardiac Transplantation, Cardiovascular Department, Rasoul Akram General Hospital, Iran University of Medical Sciences, Tehran, Iran; 6 Health Human Resources Research Centre, School of Health Management and Information Sciences, Shiraz University of Medical Sciences, Shiraz, Iran

**Keywords:** Cost-effectiveness, Heart failure, B-type natriuretic peptide, Markov model

## Abstract

**Background:**

B-type natriuretic peptide (BNP) is commonly used as a diagnostic method for patients with heart failure. This study was designed to evaluate the cost-effectiveness of BNP compared to standard clinical assessment in outpatients with heart failure with reduced ejection fraction (HFrEF) in Tehran, Iran.

**Methods:**

This study was a cost-effectiveness analysis carried on 400 HFrEF outpatients > 45 years who were admitted to Rasoul Akram General Hospital of Tehran, Iran. A Markov model with a lifetime horizon was developed to evaluate economic and clinical outcomes for BNP and standard clinical assessment. Quality-adjusted life-years (QALYs), direct, and indirect costs collected from the patients.

**Results:**

The results of this study indicated that mean QALYs and cost were estimated to be 2.18 QALYs and $1835 for BNP and 2.07 and $2376 for standard clinical assessment, respectively. In terms of reducing costs and increasing QALYs, BNP was dominant compared to standard clinical assessment. Also, BNP had an 85% probability of being cost-effective versus standard clinical assessment if the willingness to pay threshold is higher than $20,800/QALY gained.

**Conclusion:**

Based on the results of the present study, measuring BNP levels represents good value for money, decreasing costs and increasing QALYs compared to standard clinical assessment. It is suggested that the costs of the BNP test be covered by insurance in Iran. The result of the current study has important implications for policymakers in developing clinical guidelines for the diagnosis of heart failure.

**Supplementary Information:**

The online version contains supplementary material available at 10.1186/s12962-021-00334-z.

## Background

Heart failure (HF) is a significant cause of mortality, morbidity, and rehospitalization in developing countries. Despite the improvement and promotion of modern diagnostic and therapeutic strategies, HF remains an important health problem. In heart failure, the heart cannot pump enough blood to sustain blood flow to meet the body’s requirements for blood and oxygen [1–5]. The main causes of heart failure include coronary artery disease, high blood pressure, atrial fibrillation, valvular heart disease, excess alcohol use, and infection. Based on the ability of the left ventricle to contract, or to relax, there are two types of left ventricular heart failure: heart failure with reduced ejection fraction (HFrEF), and heart failure with preserved ejection fraction (HFpEF) [6].

The national burden of disease study in Iran showed the burden of cardiovascular disease will increase sharply in Iran post-2021, primarily due to the aging population [7]. Furthermore, in Iran, 79% of deaths related to chronic diseases are attributed to cardiovascular disease [8–11]. B-type Natriuretic Peptide (BNP) is a cardiac neurohormone released from the left ventricles in response to abnormal loading condition and is predictive of left-ventricular function and of prognosis [12–16]. Treatment intervention for patients with HF is usually based on clinical assessment [17]. Based on the guideline for the management of chronic heart failure in Iran, heart failure is confirmed by obtaining an accurate and complete medical history, clinical examination, and diagnostic tests. For all patients with heart failure with an ejection fraction less than 40%, Renin-angiotensin system inhibitor, in addition to beta-blocker, is recommended to reduce hospitalization due to heart failure or premature death. An electrocardiogram (ECG) is also advised to detect heart rhythm, heart rate, and other related disorders [18].

Recent meta-analyses have demonstrated that BNP-guided therapy did not decrease mortality but decreased HF hospitalization [19]. Guidelines from the American College of Cardiology Foundation (ACCF) and the American Heart Association (AHA) recommend the use of BNP or NT-proBNP as a class I indication in the diagnosis of heart failure in ambulatory patients with dyspnea [20] however, limited number of studies have been performed on the cost-effectiveness of BNP [21].

Cost-effectiveness analysis is a comparative analysis of two or more interventions based on their costs and effectiveness. Given the limited resources in the health system of developing countries, selecting and implementing cost-effective interventions can improve allocative efficiency [22, 23]. Our previous study, using a systematic review, indicated that use of BNP in patients with heart failure may reduce costs and increase quality-adjusted life years (QALYs) [24]. However, these previous studies designed and conducted in high-income countries from the perspective of the health system and provider, thus it is of limited relevance to the Iranian context. We have therefore assessed the cost-effectiveness of BNP compared to standard clinical assessment in patients with heart failure in Iran from the societal perspective. The results of this study can assist policymakers to select cost-effective interventions for patients with heart failure.

## Methods

### Study design and study population

This study was a cost-effectiveness analysis conducted in patients who were admitted to Rasoul Akram General Hospital of Tehran, Iran for heart failure.

In this study, 400 HFrEF outpatients > 45 years were followed up from August 2018 to August 2019. Patients were divided into two groups: standard clinical assessment group and BNP group. Standard clinical assessment of HF included medical history and physical examination. In the BNP arm, adjunctive testing included in addition to medical history and physical examination, with BNP measured each month from the time of admission. In this study we have excluded patients with factors influencing the BNP level including patients with high and low body mass index, glomerular filtration rate < 50 mL/min/1.73m^2^, cerebrovascular disease, pulmonary embolism and, atrial fibrillation or any acute inflammation.

**Fig. 1:**
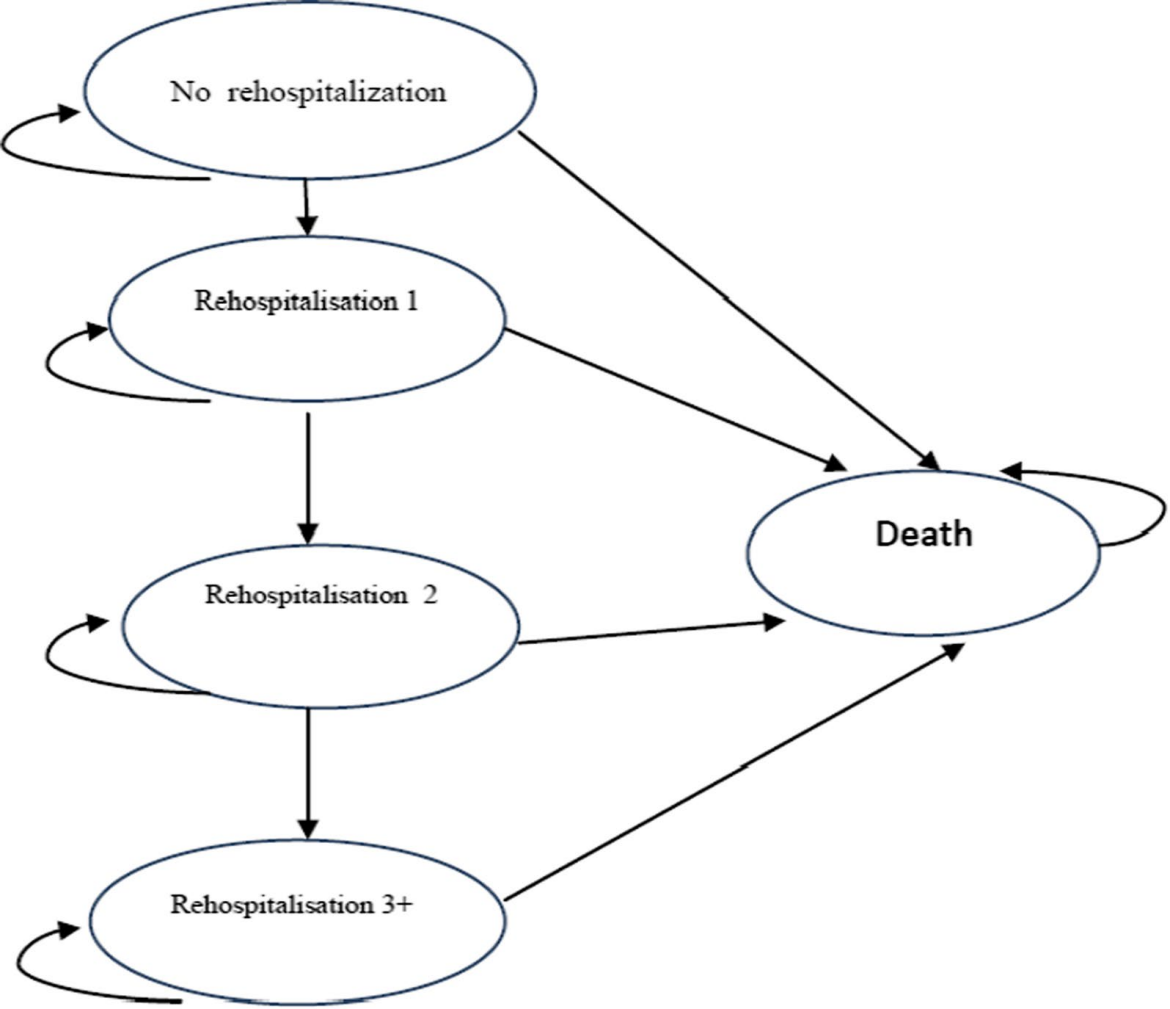
Schematic diagram of Markov model. In this diagram, ovals represent health states; arrows represent all possible transitions between health states. During each month, patients stay in the current health state without hospitalization or are readmitted and move to the next state

A five-state Markov model was developed to examine the economic effect of the clinical outcomes using hypothetical cohort of 1000 HF patients over a lifetime horizon (Fig. 1). This model was formerly validated by Delea et al. to examine the cost-effectiveness of carvedilol [25]. Given the time horizon was more than one year, costs and QALYs were discounted with an annual rate of 5.8% [26] and 3%, respectively [27]. Health states within the model based on the number of previous HF hospitalizations. The number of rehospitalisations is a valid proxy for disease progression in patients with HF [28]. During each month, patients stay in the current health state without hospitalization or are readmitted and move to the next state. The following Markov assumption were used in this study: transitions between different health states occur in one-month cycles, the distribution of patients in the model begins from the state of no rehospitalization, deaths due to natural factors in each cycle of the model based on age-specific mortality rates may occur for any reason, each patient can stay in the same states or move to the next states.

### Model parameters

We used four sets of parameters in the Markov model (Tables 1 and 2); the sources of these data are described as follows:

**Table 1:** Input parameters used in economic model

Variable	Mean	SD	Distribution	Source
Prob NRH to NRH	0.168	0.37	Beta	Göhler et al. [30]
Prob NRH to D	0.067	0.24	Beta	Göhler et al. [30]
Prob RH1 to RH1	0.213	0.36	Beta	Göhler et al. [30]
Prob RH1 to D	0.075	0.025	Beta	Göhler et al. [30]
Prob RH2 to RH2	0.268	0.37	Beta	Göhler et al. [30]
Prob RH2 to D	0.085	0.32	Beta	Göhler et al. [30]
Prob RH3 to D	0.095	0.33	Beta	Göhler et al. [30]
RR	0.81	5	LN	Pufulete et al. [16]
CostNRH_B	$309	67	Gamma	Calculated
Cost RH1_B	$446	100	Gamma	Calculated
Cost RH2_B	$585	175	Gamma	Calculated
Cost RH3_B	$861	249	Gamma	Calculated
Cost NRH_CA	$272	65	Gamma	Calculated
Cost RH1_CA	$548	153	Gamma	Calculated
Cost RH2_CA	$824	247	Gamma	Calculated
Cost RH3_CA	$1100	341	Gamma	Calculated
Utility NRH	0.85	0.23	Beta	Calculated
Utility RH1	0.828	0.25	Beta	Calculated
Utility RH2	0.809	0.26	Beta	Calculated
Utility RH3	0.777	0.24	Beta	Calculated

SD, standard deviation; Prob, probability; NRH, no rehospitalization; RH1, rehospitalization 1; RH2, rehospitalization 2; RH3, rehospitalization3; B, B-type natriuretic peptide (BNP); CA, clinical assessment; RR, relative risk; LN, Log-Normal

**Table 2:** All cause mortality rate by age in Iran

Age (range)	45–49	50–54	55–59	60–64	65–69	70–74	75–79	80–84	85 +
Mortality rate (%)	0.2	0.4	0.55	0.95	1.85	3.55	7.4	11.65	21.8

### Quality of life

In the present study, QALYs were considered as the effectiveness measure and the utility of standard clinical assessment was measured with the EQ-5D-3Lquestionnaire. This questionnaire consisted of five dimensions such as mobility, self-care, pain/discomfort, and anxiety/depression [23]. The weights of this questionnaire have been previously estimated for Iran [29]. First, using the weighted scores, the utility (between zero and one) was estimated and then the QALYs were calculated by multiplying the amount of utility in the duration of time spent in the health state. 200 patients were interviewed in each treatment group and answered the EQ-5D questionnaire. The data was collected in this part of the study by interviewing the patient. At the beginning of each interview, the overall goal of the interview was explained to each patient separately. Patients who were interested in participating in the study answered the questions voluntarily.

### Cost

Resource use was taken from the patient-level data and a bottom-up approach was applied to estimate the cost. The cost year was 2019 and the costs were identified and measured from the societal perspective. These items included medical and non-medical direct costs and indirect costs (lost productivity). 200 patients were interviewed in each treatment group and completed a data collection form. The data collection form was used to collect data, consisting of two parts: the first part collected demographic data of the patients and the second section collected information about the costs of physician visits, heart medications, BNP test, diagnostic and laboratory services, hospitalization and rehospitalization, nursing, special equipment, private service, accommodation and traveling. The costs of productivity loss due to heart failure were estimated by employing the human capital approach [30].

### Transitional probabilities

The transitional probabilities of different health states and probabilities of HF-related deaths were taken from Gohler’s study [28]. To include other-cause mortality, we used the probabilities from age-specific Iranian life tables (Table 2) [31].

### Relative risk

We used a relative risk in the model to adjust a baseline risk. The Relative risk of serial BNP for BNP vs standard clinical assessment (Table 1) was derived from the meta-analysis that was recently performed by Pufuletein et al. 2018. In the Pufuletein’s study, the effectiveness of 14 eligible randomized controlled trials related to serial BNP were examined [19].

### Cost-effectiveness analysis

Expected costs, QALYs and incremental cost-effectiveness ratio (ICER) were calculated. ICER was defined as the ratio of the difference between the costs of two alternatives to the difference between their effectiveness [22]. In addition, 95% confidence intervals (CIs) were calculated for costs and QALYs using the non-parametric bootstrapping approach [32].

### Sensitivity analysis

Deterministic sensitivity analysis (tornado diagram) and probabilistic sensitivity analysis (PSA) was performed. In the probabilistic sensitivity analysis, probability distributions for the parameters were selected and Monte Carlo simulation was performed using 1000 samples. PSA results were indicated using the cost-effectiveness acceptability curves and incremental cost-effectiveness scatter plot [33]. We also performed two scenario analyses. We first used EQ-5D values from Emrani’s study [34]. Our second scenario included different time horizon (10 and 20 years).

## Results

The results showed that the mean cost of BNP and standard clinical assessment were $682 and $649, respectively, in which the highest and lowest mean costs per patient were the direct medical costs and direct non-medical costs. The major costs in BNP and standard clinical assessments were related to the costs of medications used ($255 and $196, respectively) and diagnostic tests ($189 and $141, respectively) (Additional file 1). The result also indicated compared with standard clinical assessment; BNP patients had more significant improvements in the dimensions of mobility, self-care, and usual activities. (Additional file 2).

The expected QALYs and costs for patients who receive BNP and standard clinical assessment are shown in Table 3. According to this table, mean QALYs and cost were estimated to be 2.18 and $1835 for BNP and 2.07 and $2376 for standard clinical assessment, respectively. Although the incremental QALYs (0.11) and cost saving ($541) are relatively small, it seems that BNP is cost-effective compared to standard clinical assessment.

**Table 3:** The result of cost-effectiveness analysis

Strategy	Cost (USD)	QALYs	$\Delta \mathrm{ C}$	$\Delta \mathrm{ QALYs}$	BNP vs. Standard clinical assessment
BNP	1835	2.18	− 541	0.11	Dominant
Standard clinical assessment	2376	2.07			

QALYs, quality-adjusted life years; $\Delta \mathrm{ C}$, cost difference; $\Delta \mathrm{ QALYs}$, QALY difference

**Fig. 2:**
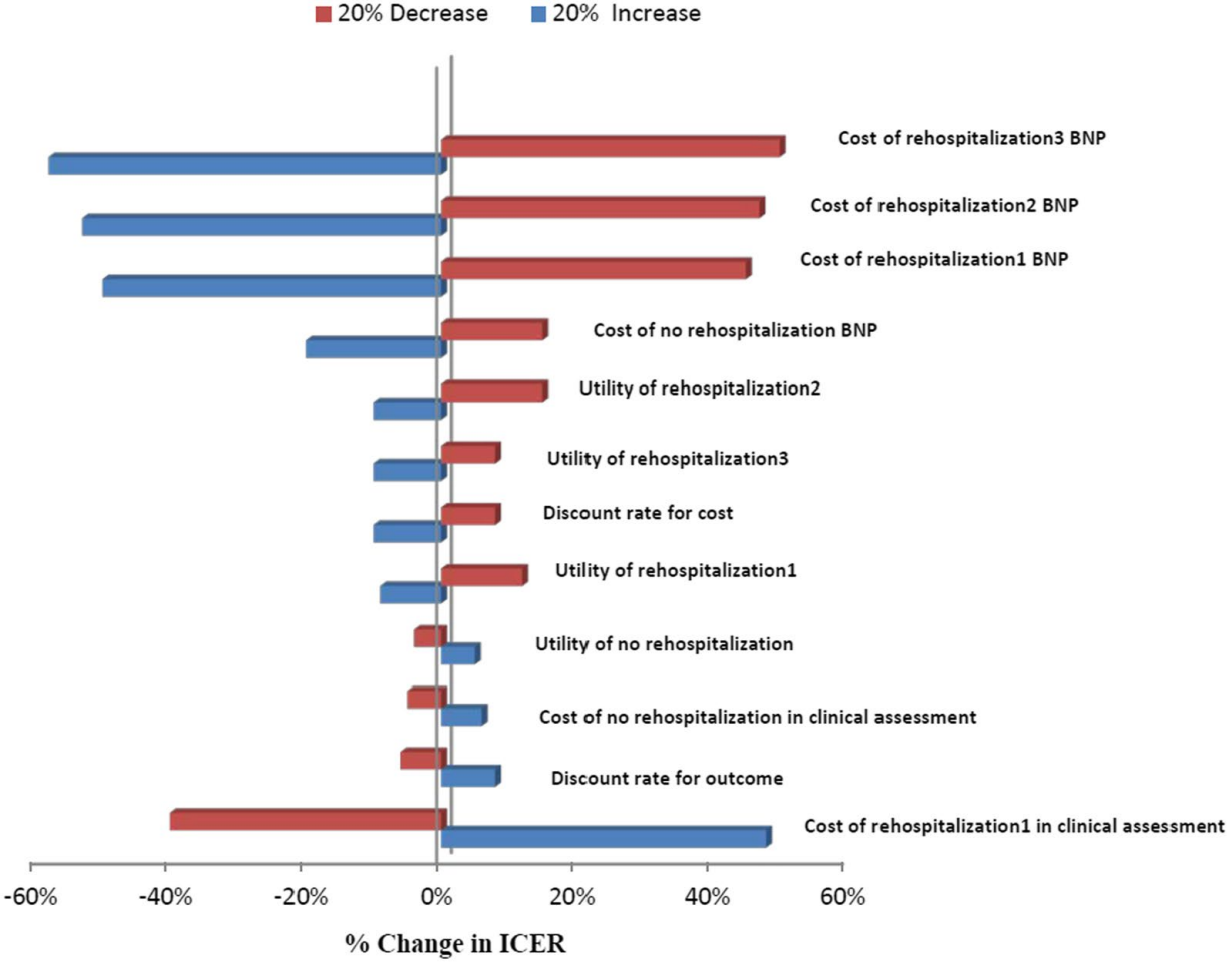
Tornado diagram for one-way sensitivity analysis. The diagram indicated that results of one-way sensitivity analysis. The value of each variable was increased and decreased by 20% and the results are shown by the Tornado diagram. The ICER had the highest sensitivities to the increase in the cost of rehospitalization3 in the BNP arm

**Fig. 3:**
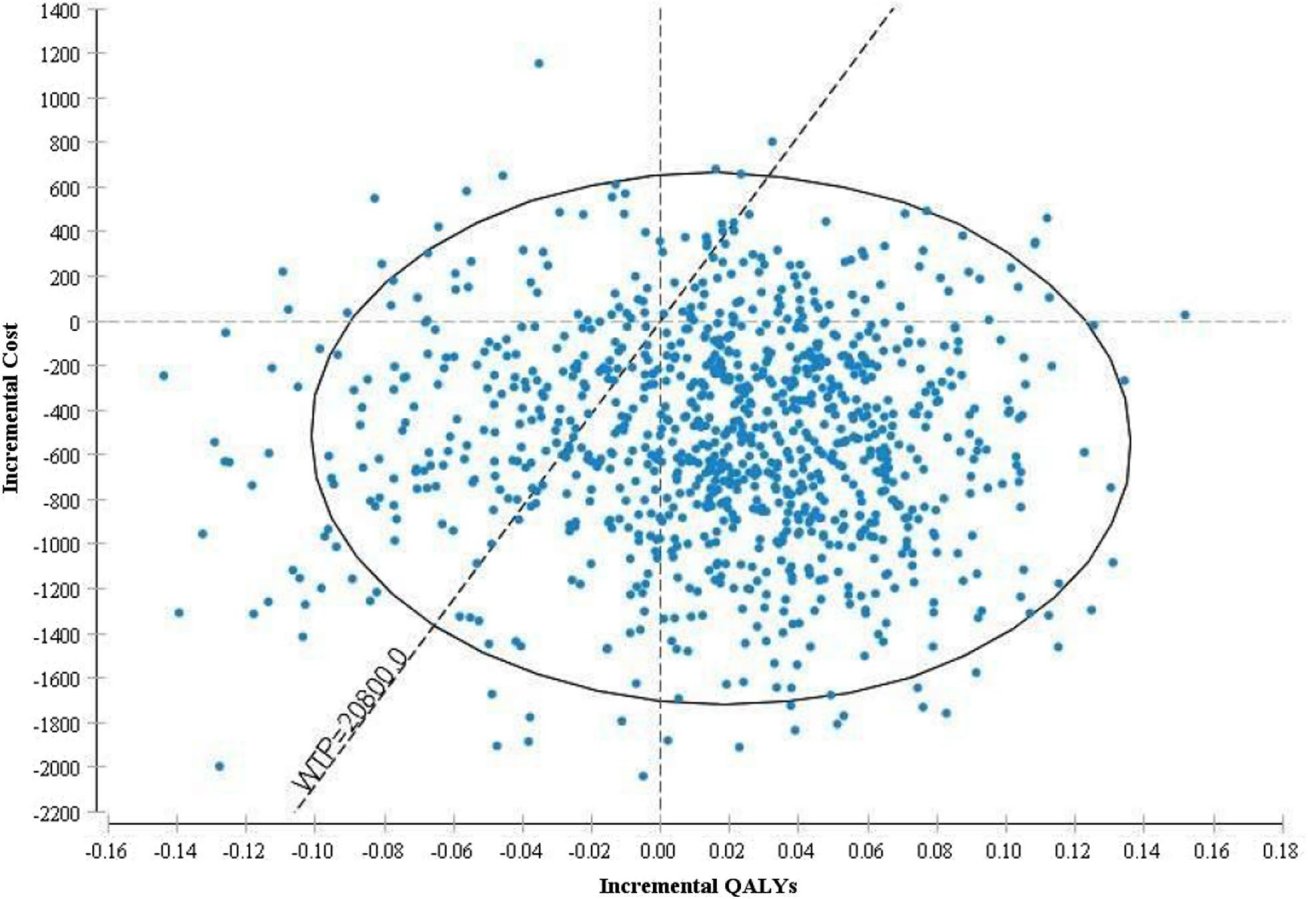
The results of the probabilistic sensitivity analysis. Each point indicates the differences in the costs and effectiveness of BNP vs. standard clinical assessment. The results showed that BNP was more cost-effective than standard clinical assessment with a maximum willingness to pay threshold of USD20800

**Fig. 4:**
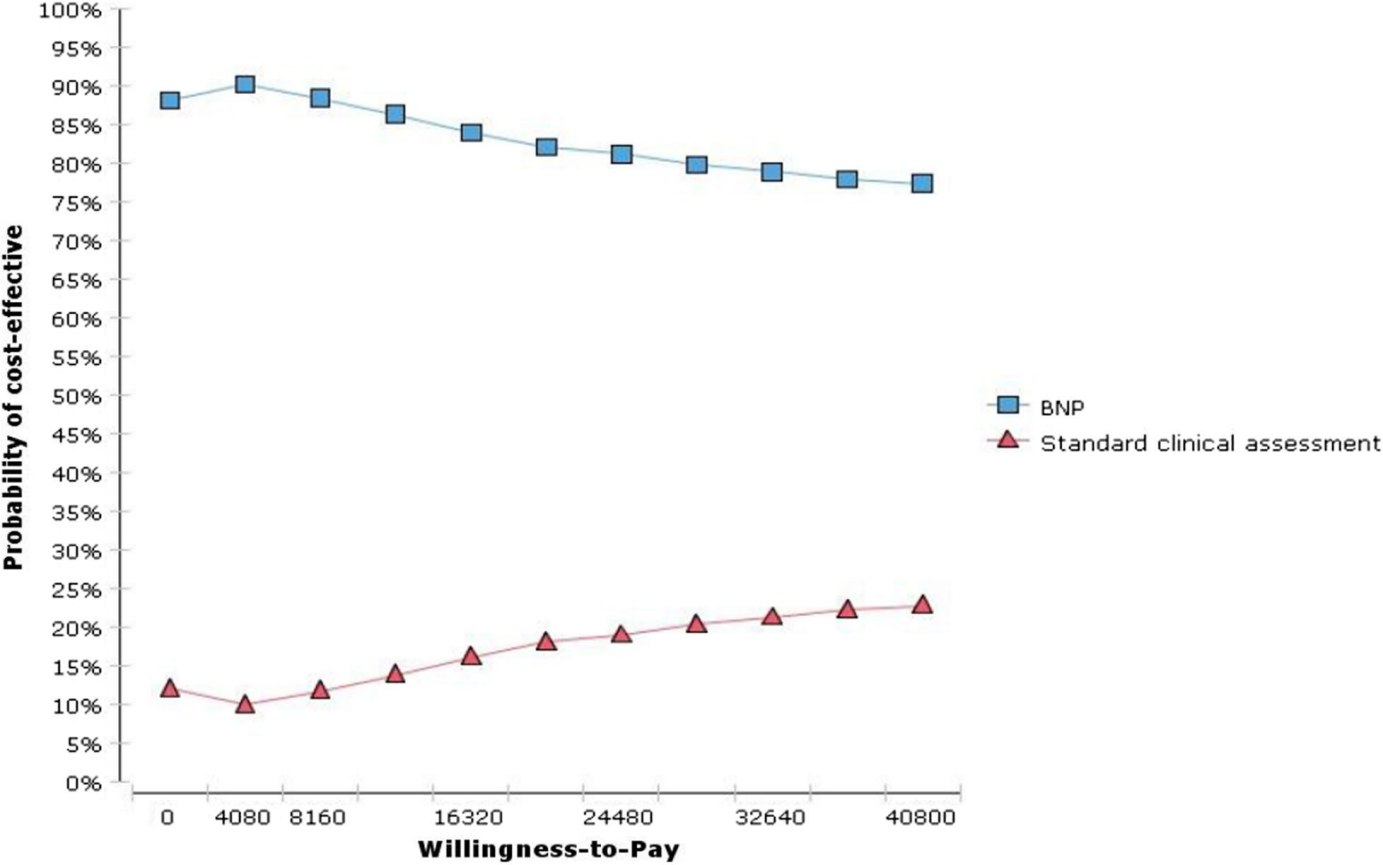
Cost-effectiveness acceptability curve. The curve shows that BNP compared with standard clinical assessment was more cost-effective at the majority of willingness to pay thresholds. BNP has an 85% chance of being cost-effective versus standard clinical assessment with thresholds higher than USD20800

Cost-effectiveness results in different age groups are shown in Table 4. BNP was dominant in the 45–55 years age group and cost-effective in 55–65 and 65–75 years age groups. Given that any economic evaluation study is associated with uncertainty, in this study, the robustness of the result was examined using one-way and probabilistic sensitivity analysis. In one-way sensitivity analysis, the value of each variable was increased and decreased by 20% separately and a tornado diagram was generated to identify the individual parameters with the greatest impact on the outcome. Based on the tornado diagram in Fig. 2, changes in most of the input parameters had few effects on the incremental cost-effectiveness ratio. Moreover, The ICER had the highest sensitivities to the increase in the cost of rehospitalization3 in the BNP arm. Figure 3 shows the results of the probabilistic sensitivity analysis using Monte Carlo simulation of incremental costs and QALYs of BNP vs. standard clinical assessment. For each of the 1000 simulations, values for parameters were randomly selected from their probability distributions. The results showed that BNP was more cost-effective than standard clinical assessment with a maximum willingness to pay threshold of USD20800 calculated based on WHO method (three times of per capita GDP, USD20800) [35]. In Fig. 4, cost-effectiveness acceptability curve (CEAC) showed that BNP compared with standard clinical assessment, was more cost-effective in the majority of willingness to pay so that BNP has an 85% probability of being more cost-effective in the thresholds higher than USD20800. Table 5, indicated the results of the scenario analysis. Based on this table, the most influential parameter was the use of EQ-5D values from Emrani’s study. Nonetheless, the BNP remained cost-effective in all scenario analyses.

**Table 4:** Cost-effectiveness results in three patient subgroups

Subgroup	BNP		Standard clinical assessment		Incremental cost per QALY gained (BNP vs. standard clinical Assessment)
	Cost (USD)	QALYs	Cost (USD)	QALYs	
45–55 years	$1100	2.07	$1280	1.24	Dominant
55–65 years	$1540	1.65	$1400	1.35	USD 466
65–75 years	$2700	1.24	$2050	1.02	USD 2954

BNP, B-type natriuretic peptide; QALYs, quality-adjusted life-years; USD, United States Dollars

**Table 5:** Scenario analysis

Strategy	Cost (USD)	QALYs	$\Delta \mathrm{ C}$	$\Delta \mathrm{ QALYs}$	BNP vs. Standard clinical assessment
Scenario 1: EQ-5D values from Emrani study					
BNP	1835	1.9	− 541	0.12	Dominant
Standard clinical assessment	2376	1.78			
Scenario 2: time horizon 10 years					
BNP	1805	2.15	− 551	0.15	Dominant
Standard clinical assessment	2356	2			
Scenario 2: time horizon 20 years					
BNP	1832	2.18	542	0.11	Dominant
Standard clinical assessment	2374	2.07			

## Discussion

To our knowledge, this study was the first cost-effectiveness analysis related to diagnostic interventions in patients with heart failure in Iran. In the present study, we directly estimated cost and QALYs using patient-level data instead of using data from the published literature. In most studies, the utility value was taken from a literature review, which could lead to misestimating of QALYs.

Based on the results, it seems that the use of BNB may reduce cost compared to the standard clinical assessment and improve QALYs. Faster diagnosis of heart failure with BNP can prevent from signs and symptoms (such as, shortness of breath with activity, fatigue and weakness, rapid or irregular heartbeat, swelling of the belly area (abdomen), difficulty concentrating or decreased alertness) and increase patients utility. Other studies confirm the results of the present study. For example, Moertl et al. indicated that the measurement of BNP level compared with the usual care and multidisciplinary care reduced costs and increased QALY [36]. Laramée et al. found that the serial BNP measurement by a specialist was the most cost-effective option compared to clinical assessment and usual care, but it is not cost-effective in people aged over 75 years of age [17]. In a similar study, Mohiuddin et al. indicated that BNP-guided care in younger patients with heart failure and reduced ejection fraction was more cost-effective than the clinical care [21]. In a similar study, Morimoto et al. showed that BNP was a cost-effective alternative to the clinical care and increased QALY and decreased costs [37]. Siebert et al. showed that the use of BNP reduced the use of echocardiography and initial hospitalization by 58% and 13%, respectively [38]. Pufulete et al. indicated that the efficacy of BNP-guided therapy in specialist HF clinics is uncertain and in the cost-effectiveness model, in patients aged < 75 years with HFrEF or HFpEF, BNP-guided therapy improves median survival with a small QALY gain but higher lifetime costs [39].

In addition, the sensitivity analyses show that there is a large amount of uncertainty associated with these results and that the results are highly sensitive to a number of key inputs (the cost values in particular). Given that both BNP and clinical assessment can be performed in hospitals of Iran, the results of this study can be generalized to other Iranian hospitals. As to other countries, other factors such as the degree of costs covered by insurance organizations, the patients’ willingness to pay, the incidence and prevalence of disease, difference in clinical guidelines, relative prices, reimbursement system and threshold should be considered. The few numbers of studies have been performed on the cost-effectiveness of diagnostic methods in patients with heart failure in developing countries, while the majority of people with heart diseases are living in countries with middle and low income and it needs to be performed more economic evaluation researches in this field in developing countries. This study had some limitations. First, the efficacy of diagnostic methods in patients with heart failure is mainly dependent on factors such as the sensitivity and specificity of laboratory tests and the period of patient follow-up. Second, we used a hypothetical cohort model; the real condition may be different from our model. However, the strength of our study was to use societal perspective, measure the direct and indirect cost of the patient, and use validated weight of utility for the Iranian population.

According to results of the current study, measuring BNP levels probably represents good value for money. It reduced costs and increased QALYs compared to standard clinical assessment. Given the result of this study, it is suggested that the cost of the BNP test be covered by insurance because of its high costs for heart failure patients. Moreover, it suggested that when developing clinical guidelines for the diagnosis of heart failure, policymakers should approve monitoring BNP levels in heart failure patients in Iran.

## Supplementary Information

**Additional file 1.** Direct and Indirect Costs in BNP and standard clinical assessment.

**Additional file 2.** Mean of disutility in study groups.

## Data Availability

All related data were displayed in the manuscript. Further information regarding the data can be obtained by contacting the corresponding authors.

## Abbreviations

BNP: B-type natriuretic peptide
HfrEF: Heart failure with reduced ejection fraction
QALYs: Quality-adjusted life years
PSA: Probabilistic sensitivity analysis
CEAC: Cost-effectiveness acceptability curve
ICER: Incremental cost-effectiveness ratio
ACCF: American College of Cardiology Foundation
HFpEF: Heart failure with preserved ejection fraction
AHA: American Heart Association
NT-proBNP: N-terminal pro-B-type natriuretic peptide
