# Supplementary material for: The cost-effectiveness of B-type natriuretic peptide-guided care in compared to standard clinical assessment in outpatients with heart failure in Tehran, Iran
Source: Cost Eff Resour Alloc. 2021 Dec 23;19:81. doi: 10.1186/s12962-021-00334-z (PMC8705161; doi:10.1186/s12962-021-00334-z)
Supplement: Supplementary file 1 — Additional file 1. Direct and Indirect Costs in BNP and standard clinical assessment. [file 12962_2021_334_MOESM1_ESM.docx]

**Additional file 1. Direct and Indirect Costs in BNP and standard clinical assessment**

| Type of costs | Type of costs | BNP | | Standard clinical assessment | |
| --- | --- | --- | --- | --- | --- |
|  |  | Mean (USD) | % | Mean (USD) | % |
| Direct medical costs | Medications and drugs | 255 | 44 | 196 | 37 |
|  | Diagnostic test | 189 | 32 | 141 | 26 |
|  | Outpatient cost | 15 | 3 | 12 | 2 |
|  | Inpatient cost | 126 | 22 | 185 | 35 |
|  | Total | 585 | 100 | 534 | 100 |
| Direct non-medical costs | Accommodation | 23 | 53 | 24 | 51 |
|  | Transportation | 20 | 47 | 23 | 49 |
|  | Total | 43 | 100 | 47 | 100 |
| Indirect costs | Patient companions’ absenteeism due to patient care | 40 | 74 | 47 | 69 |
|  | Absence from work due to the disease | 14 | 26 | 21 | 31 |
|  | Total | 54 | 100 | 68 | 100 |
| Total |  | 682 | 100 | 649 | 100 |
