# Supplementary material for: The cost-effectiveness of B-type natriuretic peptide-guided care in compared to standard clinical assessment in outpatients with heart failure in Tehran, Iran
Source: Cost Eff Resour Alloc. 2021 Dec 23;19:81. doi: 10.1186/s12962-021-00334-z (PMC8705161; doi:10.1186/s12962-021-00334-z)
Supplement: Supplementary file 2 — Additional file 2. Mean of disutility in study groups. [file 12962_2021_334_MOESM2_ESM.docx]

**Additional file 2. Mean of disutility in study groups**

| Dimensions of EQ-5D | BNP | standard clinical assessment | P-value |
| --- | --- | --- | --- |
| Mobility | 0.047 | 0.133 | 0.03 |
| Self- care | 0.041 | 0.086 | 0.02 |
| Usual activities | 0.070 | 0.091 | <0.001 |
| Pain/discomfort | 0.101 | 0.116 | 0.12 |
| Anxiety/depression | 0.090 | 0.131 | 0.19 |
